# Long-Term Occupancy Trends in a Data-Poor Dugong Population in the Andaman and Nicobar Archipelago

**DOI:** 10.1371/journal.pone.0076181

**Published:** 2013-10-15

**Authors:** Elrika D’Souza, Vardhan Patankar, Rohan Arthur, Teresa Alcoverro, Nachiket Kelkar

**Affiliations:** 1 Oceans and Coasts Program, Nature Conservation Foundation, Mysore, Karnataka, India; 2 Centre d'Estudis Avançats de Blanes (Spanish National Research Council), Blanes, Girona, Spain; Università degli Studi di Napoli Federico II, Italy

## Abstract

Prioritizing efforts for conserving rare and threatened species with limited past data and lacking population estimates is predicated on robust assessments of their occupancy rates. This is particularly challenging for elusive, long-lived and wide-ranging marine mammals. In this paper we estimate trends in long-term (over 50years) occupancy, persistence and extinction of a vulnerable and data-poor dugong (*Dugong dugon*) population across multiple seagrass meadows in the Andaman and Nicobar archipelago (India). For this we use hierarchical Bayesian dynamic occupancy models accounting for false negatives (detection probability<1), persistence and extinction, to two datasets: a) fragmentary long-term occurrence records from multiple sources (1959–2004, n = 40 locations), and b) systematic detection/non-detection data from current surveys (2010–2012, n = 57). Dugong occupancy across the archipelago declined by 60% (from 0.45 to 0.18) over the last 20 years and present distribution was largely restricted to sheltered bays and channels with seagrass meadows dominated by *Halophila* and *Halodule* sp. Dugongs were not found in patchy meadows with low seagrass cover. In general, seagrass habitat availability was not limiting for dugong occupancy, suggesting that anthropogenic factors such as entanglement in gillnets and direct hunting may have led to local extinction of dugongs from locations where extensive seagrass meadows still thrive. Effective management of these remnant dugong populations will require a multi-pronged approach, involving 1) protection of areas where dugongs still persist, 2) monitoring of seagrass habitats that dugongs could recolonize, 3) reducing gillnet use in areas used by dugongs, and 4) engaging with indigenous/settler communities to reduce impacts of hunting.

## Introduction

Many species of marine mammals have undergone major declines in the world’s oceans in the recent past due to threats ranging from habitat loss, interactions with fisheries and hunting, their numbers now restricted to small populations across their range [1], [2]. The effective management of these remnant populations requires an understanding of factors underlying long-term changes leading to their present distribution. For these typically long-lived, wide-ranging and elusive species [2], obtaining reliable population estimates across large spatiotemporal scales can be a considerable challenge [3], [4].

Recent advances in occupancy-based modeling provide a probabilistic description of species’ distribution across large spatial scales as well as a useful framework to identify factors influencing distributional changes in marine species over time [3], [5], [6], [7]. Long-term studies can help identify sites where rare and threatened marine species have persisted or become extinct, and can be useful for prioritizing conservation strategies [8]. But only a few studies, predominantly on terrestrial species, have attempted to describe long-term occupancy dynamics with robust, quantitative methods [9], [10].

In the case of rare marine mammals, these estimates can be difficult to obtain at large scales, as they are almost always, subject to sampling error, related to spatial coverage and false negatives (i.e. imperfect detection) [11]–[13]. Another common problem with rare and threatened marine mammals is the poor availability of data on their past occurrence, making conservationists rely largely on anecdotal or fragmentary information that is viewed as “less than robust”.

Historical records remain among the few sources of information on most rare species [14]. These data are often too fragmentary to coherently assess and identify trends and timing of population decline [14], [15]. In the absence of ‘better' data, however, it is vital to explore these sources of information to develop current conservation strategies for endangered marine mammals, as they may conceal important clues about past declines [16]. Advances in analysis techniques, including occupancy models, allow us to make the best use of such data and given biological assumptions, to infer trends in the distributional status of species [10].

Apart from issues of data quality, effective sampling strategies can also be difficult to devise. Oceanographic patterns, topography, depth and nutrient flows make marine environments highly dynamic [17], adding to difficulties in conducting distribution surveys with the assumption of habitat stability during survey periods [6]. These problems are aggravated by logistical and technical constraints in covering large areas, which often limit sampling coverage, particularly in developing countries where these species occur [18]. These challenges result in limited knowledge of occupancy dynamics of threatened marine mammals across their range [19].

The dugong (*Dugong dugon*), classified as Vulnerable (IUCN, 2012), is a case in point, having shown considerable reductions across many seascapes of the Indo-Pacific region [20]. Yet, data on population status and distribution are still scarce from many regions, although they represent an important part of the current global distribution. As a result, our understanding of the ecology and distribution of dugongs is informed largely from well-studied regions like Australia, where their densities are typically higher than most other regions in the animal’s present range [21]. In South Asia, dugong numbers have shown declines and are currently restricted to small, localized populations, the status of which is poorly known [22], [23]. In India, the population occurring in the Andaman and Nicobar (A&N) archipelago might form an important connecting link between Southeast Asian (Indochinese) dugong populations (e.g. Thailand and Myanmar [23]–[25]). Despite the global importance of this population, information from the region is scarce and no comprehensive surveys of dugong distribution have been carried out. Most historical records of dugong occurrence from this archipelago are based on opportunistic sightings and mortality reports [26]. These records indicate that dugongs were apparently common here until the 1960s. Das and Dey [27] estimated approximately 40 dugongs in 1994-95, based on a compilation of chance encounters reported by fishers and dive operators, but this number was based on fairly limited survey effort and coverage.

The sparseness of information from this population has been mainly due to the considerable difficulties in surveying the archipelago. Apart from their remoteness, most A&N islands are uninhabited or difficult to access, being protected as indigenous tribal areas by the Andaman & Nicobar Islands Protection of Aboriginal Tribes Regulation [28]. Additionally, ship or aerial surveys are not feasible given the low visibility and the high costs of carrying out large-scale surveys. Under these conditions, land- and boat-based sampling [29] offers logistically easier and cheaper alternatives. Even in the absence of direct sightings, dugongs, being considerably dependent on seagrass meadows for their forage requirements, leave unmistakable feeding trails that last for several days [30], allowing us to reliably determine recent meadow usage. These surveys, if conducted in a systematic spatial sampling framework, yield data that can be analyzed effectively with occupancy models. In addition, these models can prove helpful in estimating past distribution dynamics, by addressing issues of imperfect detection inherent to available historical records.

In this paper we use historical records and systematic archipelago-wide surveys in two separate model sets, to describe 1) long-term trends in dugong distribution and 2) identify factors potentially affecting current dugong occupancy within the A&N archipelago. We analyzed past records from multiple seagrass meadows (over 45 years) with Bayesian hierarchical, dynamic occupancy models based on a detection and non-detection framework. To assess short-term (current) occupancy dynamics we conducted comprehensive surveys of dugong presence/absence in seagrass meadows over three consecutive years. Based on our results, we discuss applications of occupancy-based approaches to assess long-term distribution dynamics of small populations of threatened marine mammals. Finally, we demonstrate how these estimates can help in developing and prioritizing site-specific conservation strategies for dugongs in the A&N archipelago.

## Materials and Methods

### Ethics statement

The dugong is listed under Schedule I of the Indian Wildlife (Protection) Act, 1972 and hence we obtained all necessary permits from the Chief Wildlife Warden, Department of Environment and Forests, Port Blair, India to study the species and its habitats. As our research did not involve handling or collection of animals, specific animal handling permits were not sought and ethical clearance for did not apply. Part of the study was carried out in and around protected areas, national parks, sanctuaries and tribal reserves. For this purpose, entry permits were obtained from the Department of Environment and Forests and the Andaman and Nicobar Islands Administration.

Our work with human communities adhered to all standard scientific ethical norms and we received due clearance from Nature Conservation Foundation's human ethics committee for this work. The informants in our study included members of tribal and settler communities. Apart from settlers, only members of the Nicobarese tribal community were part of our informant network due to restrictions on entry and interactions with other indigenous communities. Levels of literacy are very low in these communities and because written permissions were therefore not possible, our studies were conducted after verbal consent and informal permissions were obtained from local Nicobarese Tribal Council Heads. The measure for documenting the process of verbal consent was to have the consent before witnesses from the community appointed by the council head that would also accompany us during the interviews. The ethics committee of the Nature Conservation Foundation independently contacted these enlisted witnesses regarding their witnessing of verbal consent, and after such confirmation approved the process. Further, we obtained hunting records from the Nicobarese only from voluntary reporting of dugong sightings by community members themselves. All these interactions were conducted in a manner respectful of local cultural norms and traditions and in the presence of a village head.

Due to legal issues and sensitivity in discussing issues pertaining to hunting, individuals belonging to settler communities were not asked to report records of illegally hunted animals; this information was obtained only from records maintained with the Department of Environment and Forests. The ethics committee of the Nature Conservation Foundation (Mysore, India) also approved of these methods.

### Study area

The Andaman and Nicobar archipelago of India, is part of the Indo-Myanmar and Sundaland biodiversity hotspots [31], [32] between latitudes 6^o^45' N and 13^o^41' N and longitudes 92^o^12' E and 93^o^57' E in the southeastern part of the Bay of Bengal. This archipelago comprises more than 350 islands, occupying an area of 8,249 km^2^ with a total coastline of 1,962 km that includes the Andaman group (>325 islands, 24 inhabited, 6,408 km^2^) and the Nicobar group (21 islands, 13 inhabited, 1,841 km^2^ [33]). This region is significantly influenced by the southwestern and northeastern monsoons (May-December). The islands have highly diverse terrestrial and marine ecosystems, comprising evergreen and littoral mangrove forests, extensive seagrass meadows, fringing coral reefs and active volcanic islands. The islands have historically been occupied by indigenous tribes of negroid (Onge, Jarawa, Great Andamanese, Sentinelese) and mongoloid (Nicobarese, Shompen) origins [33]. These native tribes constitute only about 9% of the present population, which is dominated by recent (c.80–100 years) immigrant settlers from mainland India, Bangladesh, Sri Lanka and Myanmar. Agriculture, livestock rearing, fisheries and plantation forestry are the main occupations in the islands, and the indigenous tribes still significantly depend on minor forest produce and hunting, including ritual hunting of dugongs [34].

### Data collection

**Historical records of dugongs.** A comprehensive database of historical records of dugongs (n = 55 records of 124 individual dugongs) was collated spanning the last 50 years (1959–2009) from 40 seagrass meadow locations across the A&N archipelago (Appendix S1). Newspaper reports, occasional publications, fisheries by-catch records, reports by local forest departments, and interviews with key informants and wildlife experts were used to compile these records to represent these occurrences. The records included sightings of dead (entangled in gillnets or hunted) and live animals. As such, we expected considerable bias in naïve occupancy estimates, evidently associated with false negatives or imperfect detection (detection probability of less than 1) that historical ‘presence-only’ data are typically subject to [14], [16]. To allow for detection probabilities of dugongs to be estimated meaningfully (with relatively uniform coverage) across islands, we grouped the historical data into 3 primary periods of 15 years each (45 years, 1959–2004), further sub-dividing each primary period into three replicate periods of 5 years each. We did not use data on dugong records from 2005–2009 for historical records, as most of it came from our focused surveys at a few sites, and could have biased detection relative to other sites not surveyed in this period. We covered all sites uniformly in the current (short-term) dynamic occupancy surveys (2010–2012). This assumption was based on the possibility that at least one dugong sighting was likely to be reported from one location within a 5-year period. Given the general rarity of dugong sightings, maintaining a shorter replicate period could have led to biased assignment of probable detection as a ‘false absence’, thus leading to increased estimation of false-negatives (or Type II errors) [35], [36]. On the other hand, a longer replicate period could have led to unnecessary loss of ‘detected’ data. Based on these assumptions, we constructed a detection-non-detection matrix based on sighting records [36] for 40 meadow locations over 3 primary periods. We also assumed that dugong occupancy, persistence and extinction probabilities would not change across replicates but only across primary periods (15 years), based on calving intervals [37], [38]; movement patterns and home ranges (as estimated in tagging studies in Indonesia [39]) and our personal direct observations of three individually identified dugongs over two, four and seven years respectively. For these models, we assumed survey coverage across sites to be similar given the duration of the replicate periods. Although these assumptions appear ad hoc and still have some limitations with respect to spatial coverage, our recent field observations (regularly obtained between 2005 and 2012) suggested that they provide a reasonable idea of occupancy and detection across sites [16], [37]. The details of the assumptions and their bearing on our long-term occupancy models are given in Table 1. For each meadow we also obtained information on wave exposure and depth from bathymetry maps [40] and ground measurements. Live and dead dugong sighting records within replicate periods could not be correlated with anthropogenic threats, as threat data could not be reliably obtained for all locations over 45 years.

**Table 1:** Description of and limitations about the stated assumptions in parameters of long-term and short-term dynamic occupancy models.

Model (parameter)		Assumption		Justification	Limitations	
*Dynamic occupancy models*						
Probability of Occupancy (ψ)	Changes across 15 year periods^a^ and annually^b^		Probability of occupancy derived conditional on detection probability, estimation for short-term data better than long-term			Occupancy estimates are conservative because of negative bias in detectability, but are preferable to overestimates
Detection probability *(p)*	^a^ constant within 5-year periods, ^b^ assumed to change annually and as per method used in sighting or feeding trail detection		Model explicitly estimates detection probability, i.e. the probability of having false negatives in the data; estimation far more robust for current short-term data than for long-term data			False negatives expected to dominate the long-term datasets, so estimates of detection probability are conservative (typically with slight negative biased)
Probability of persistence/Colonization-Extinction (φ, γ)	Changes after 15 years, constant over 5-year secondary replicates; assumed to change annually^b^		Assumption based on our own field observations of 3 identified individual dugongs, and from home ranges reported by De Iongh et al. (1998)			Sheppard et al. (2007) suggest that dugong movements may be more individualistic and longer ranges may be covered, however given our observations, this seemed relatively unlikely.
Ecological covariates (*β)*	^a^ Fixed site-specific covariates (e.g.) exposure, depth that would not change at ecologically significant scales over time; ^b^ site-specific covariate data on seagrass meadows and anthropogenic threats based on annual monitoring		Covariates assumed to be static and not changing over time for long-term models; Mean and standard deviations of covariate values used over three years			Unable to use other covariates related to human disturbance, etc. for long-term models, due to gaps and missing data
Survey coverage	^a^ Data from about 60% of known extant seagrass meadows, in the absence of data on the condition of past meadows ^b^ Nearly 80-85% of the total Lakshadweep archipelago surveyed for seagrasses		Bias in parameter estimates possibly differs between sites			Model cannot account explicitly for these differences, so only locations with minimum of three data points included. Patchy sampling coverage might also negatively bias detectability, but considering the scale of the study, it is a logistical constraint

Key: ^a^ Long-term dynamic occupancy models; ^b^ Short-term dynamic occupancy models.

**Meadow persistence and current dugong occupancy.** Information on trends in dugong occupancy from long-term occupancy models was used as prior information in the current, short-term occupancy models (based on data between 2010-2012). Current occupancy was assumed to be weakly dependent on long-term occupancy, as it would provide a clear way of identifying meadow sites that have had long-term dugong persistence or recent extinction/colonization. A correspondence or increase between past and current occupancy would imply persistence or colonization, whereas a decrease would indicate local extinction. Second, we estimated relative seagrass meadow persistence for sites where past information was available over a 15-year period, comparing an earlier seagrass status report [41] with our surveys. We tracked 26 out of the total 57 surveyed seagrass meadows over a seven-year period (2005–2012) and used data on seagrass cover and community composition to categorize the meadow dynamics at each location as follows: (1 = Lost, 2 = Recently established, 3 = Dynamic changing, 4 = Stable/Persistent). We collected primary detection and non-detection data on dugongs from 57 meadows after a comprehensive survey across c. 75% shoreline area of the A&N archipelago between 2010–2012. These data were collected using two methods: i) boat-based surveys (for direct sightings) and SCUBA diving and snorkeling (for feeding signs of dugongs in meadows), and ii) sightings by reliable key informants compiled in parallel along with the time of our surveys. At each site where meadows were present, two observers scanned the surface for direct sighting of dugongs using boat transects parallel to the shoreline contours throughout the seagrass meadow and surrounding area (based on [29]). *In situ*, by snorkeling and SCUBA diving, we surveyed each meadow for dugong feeding signs along four 50 m transects distributed randomly in the meadow, covering 70–80% of the meadows for optimal coverage, adequate spacing of transects and overall efficient sampling. During our seagrass surveys we also checked for leaf-cropping signs (by dugongs or green turtles) on other species (*Thalassia hemprichii, Cymodocea rotundata, Cymodocea serrulata, Enhalus acoroides*) since it has been suggested that dugongs may also crop these species without leaving feeding trails [42]. However cropping of seagrasses was not observed on these species throughout our study, making us reasonably certain that feeding trails provided an unbiased measure of the overall extent of dugong presence at each site. Seagrass meadows surveyed had mostly sandy substrates (92%); hence the influence of sediment type on detection of feeding signs was negligible. Since we employed SCUBA and snorkeling in every seagrass meadow surveyed there was no effect of water depth on detection of feeding signs. Meadow-level occupancy data were pooled from these replicate transects within each sampling location. To avoid misidentification of feeding signs, we familiarized ourselves with characteristics of typical feeding trails (c.20 cm wide) by following feeding dugongs and examining the signs they left behind. In the same survey period we interviewed key informants who were familiar with and regularly visited each location (across 57 meadows); we compiled detection and non-detection data from their observations during the year. Replicate detections from our direct observations/feeding signs and from informant reports were recorded as ‘1' and non-detection as ‘0', to form a 1/0 matrix for each location over 3 years.

**Figure 1:**
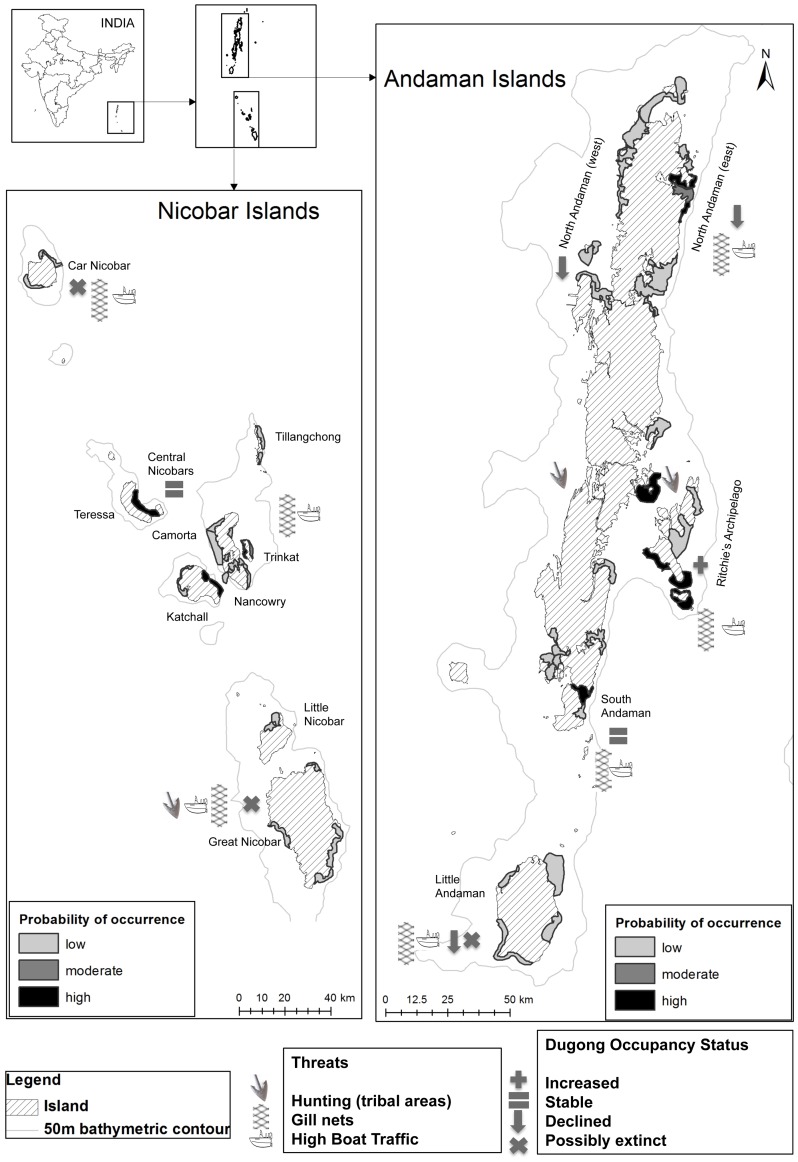
Current distribution of dugongs in the Andaman and Nicobar archipelago. Dugong occupancy (ψ: low <5%, moderate 5–30%, high 30–100%) is indicated in relation to anthropogenic threats present in different areas. Dugong presence appears mainly restricted to the Ritchie’s Archipelago, Central Nicobars and South Andaman. N.B.: The volcanic islands of Barren Island and Narcondam Island are not shown in the figure (see insets).

In each year, surveys were conducted for a fixed period of three months (Feb-May) corresponding with the observed period of highest seagrass biomass (personal observations), [43], [44] that allowed us to assume occupancy state to be closed within each year, but to change from year to year. The size of individual sampling units (locations including seagrass meadows, with seagrass cover larger than 100 m^2^ and the surrounding landscape (4–8 km^2^)) was determined based on published studies on dugong home ranges, residence time and foraging time [44], [45]. Studies have found that dugong movements are highly individualistic, and it may thus be difficult to estimate home ranges [46]. However, a clear operational definition of such a ‘home-range’ was necessary to delineate sampling units. Dugongs showed high site-fidelity and occurred within localized areas throughout the sampling period, and showed ranging behavior similar to the range sizes reported by de Iongh et al. (1998) [39]. Within each year, we assumed feeding dugongs to have stayed in the same sampling units through the short survey periods, a pattern confirmed by our long-term observations on 3 dugongs. We preferred to use estimates of movement reported by de Iongh et al. (1998) over the detailed study by Sheppard et al. (2007) [46], despite the former’s low sample size, as they reported similar herd sizes and habitat extents to our study area, perhaps due to the geographical proximity. On the other hand, Sheppard et al. (2007) reported considerably large herd-sizes, extensive habitats, large movement ranges and traveling distances, which differed from our dugong observations. Sampling units were nested under larger ‘island-groups’ within a range of 500–800 km^2^ along the 50 m bathymetry contour (Fig. 1).

We also measured meadow characteristics including depth, wave exposure, seagrass species composition, shoot density, and patchiness. Depth and wave exposure were determined using bathymetric data [40] and our ground surveys. We recorded seagrass species composition within each meadow using three quadrats of 20×20 cm^2^. Meadow composition was classified as 1 = *Halophila* sp. (*Halophila ovalis* and *Halophila minor*) dominated (95% abundance) or *Halodule* sp. dominated (95% abundance), 2 = *Halophila* sp. + *Halodule* sp. co-dominants (either species <95% abundance), 3 = *Halophila* sp. and/or *Halodule* sp. plus other species (out of *Thalassia hemprichii, Cymodocea rotundata, Cymodocea serrulata, Enhalus acoroides, Syringodium isoetifolium*); and 4 = mixed meadows without *Halophila* and *Halodule* spp. [41], [47]. We assessed meadow patchiness while looking for dugong feeding signs along 50 m strip transects, along which we recorded transitions in benthic cover (seagrass to sand/others) at a minimum interval> = 20 cm. We estimated percent seagrass cover and classified meadows with patchy (<50%) seagrass cover as fragmented, and meadows with over 50% cover as continuous. Shoot densities were estimated and tracked across 14 out of the 57 meadows.

We recorded anthropogenic disturbance with direct observations at 38 out of the total sampled meadows (57). This included 1) gillnet fishing (1 = presence of at least one gillnet in the area during the sampling period and 0 = absence of gillnets on all sampling days), 2) hunting (present/absent) based on records of legal (by tribes) or illegal hunting (by settlers) and 3) level of boat traffic observed on each sampling day (low = 0–1 boats/day, moderate = 2–5 boats/day and high = 5+ boats/day) averaged across all sampling days and years (minimum of 5 days per site).

### Data analysis

Two separate sets of dynamic occupancy models were applied to detection and non–detection data from 1) long-term (historical) records, and 2) short-term or current surveys respectively, based on standard methods developed by MacKenzie et al. (2006) (2002) [7], [37], MacKenzie and Royle (2005) [35], Royle and Kery (2007) [48], Bailey et al. (2007) [49] and Royle and Dorazio (2008) [50]. Short-term occupancy models were further modified to incorporate false-positive detection errors [36], [51], multiple detection methods [52] and spatial random effects [53] between adjacent meadows included within a larger ‘island-group’.

We estimated false-positive detection errors to account for possible misidentification of dugongs by informants. However, all informants gave highly accurate descriptions of their encounters, and we expected false-positive errors to be fairly low. We modeled long-term occupancy dynamics using the covariates ‘exposure’ and ‘depth’ at each meadow. For modeling current occupancy dynamics, ‘meadow persistence’, ‘depth’, ‘wave exposure’, and ‘seagrass species composition’ were the ecological covariates used. Due to missing data on seagrass shoot density, meadow cover and anthropogenic threats (data available from 20, 26 and 38 meadows respectively), we did not directly model effects of these covariates on occupancy. Post-hoc, we compared numbers of live and dead dugong sighting records across meadows where occupancy had declined over time, versus meadows where occupancy was estimated as stable. We also compared present-day occupancy rates at meadows from where data on anthropogenic threats were available (n = 38).

Bayesian hierarchical modeling helped us describe complex dynamic occupancy models using information from multiple sources. Estimates from historical models were used for constructing appropriate prior distributions of current occupancy, thereby linking historical occupancy with present occurrences. Detection and non-detection (1/0) data were updated using the Bayes theorem *p(θ|data)* ∝ *p(data|θ).p(θ)* where θ represented the set of model parameters (e.g. occupancy, persistence, detection), and ‘posterior’ probabilities for these parameters conditional on the actual data were estimated. Hierarchical modeling of occupancy data was based on parameter estimation at two levels: 1) the actual process of dugong occupancy and 2) the imperfect detection of this process [50]. We modeled occupancy ψ as a function of habitat and spatial random effects, specifying the model as a binomial GLM *logit*(ψ_i_ **)**<-*b0*+b1*habitat covariate [i]+*spatial effects* where the actual ‘presence or absence at a meadow *i* at time *t*’ is treated as a Bernoulli random variable *Z_it_ ∼ Bernoulli*(ψ_i_). The imperfect detection of *Z* was modeled as *mu* [*i,j,t*]<-Z [i,1]*Pr [i,j,t]; where *Pr* was the detection probability for meadow *i*, observation *j* and time *t*, and *Y* the observed fraction of sites, as *Y*_ijt_ ∼ *Bernoulli(mu [i,w,t])*.

The probabilities of occupancy ψ_i_ and detection *Pr [i,j,t]*, as well as persistence/colonization were generally described with a *Uniform (0,1)* prior distribution. Based on the quality and quantity of information available on historical occupancy at a meadow, we specified appropriate prior distributions, by scaling their variance terms as high (for anecdotal records) and relatively low (very believable records or photographs). Other than this specification, we mostly used uninformative prior distributions for intercept and slope parameters of the models, with a normal distribution centered on zero mean and high variance: *Normal (0, 1000)*. For expected positive and negative slopes, we used lognormal and normal distributions with positive or negative means; but with high variance. We conducted all analyses in R 2.15.0 and OpenBUGS 2.2.0 [54], [55]. Details of Bayesian model specification are given in Appendix S2.

## Results

### Long-term occupancy dynamics

**Figure 2:**
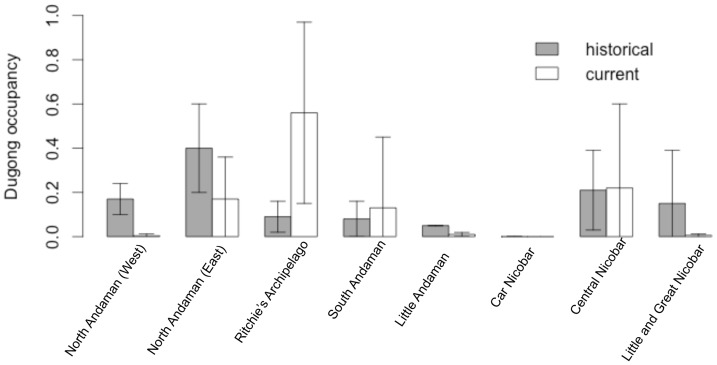
Changes in dugong occupancy (ψ) across the Andaman and Nicobar archipelago over 50 years (1959 –**2009).** Dugong occupancy (ψ) appears to have been stable in three regions: Ritchie’s Archipelago, Central Nicobars and South Andaman (0.13–0.56). Major historical declines were estimated from north Andaman (from 25% to 0.10%), Little Andaman (5% to 0.01%) and Little and Great Nicobars (20% to 0.06%). It is unclear if dugongs occurred, even in the past, around the Car Nicobar Island. Error bars indicate standard deviation.

**Figure 3:**
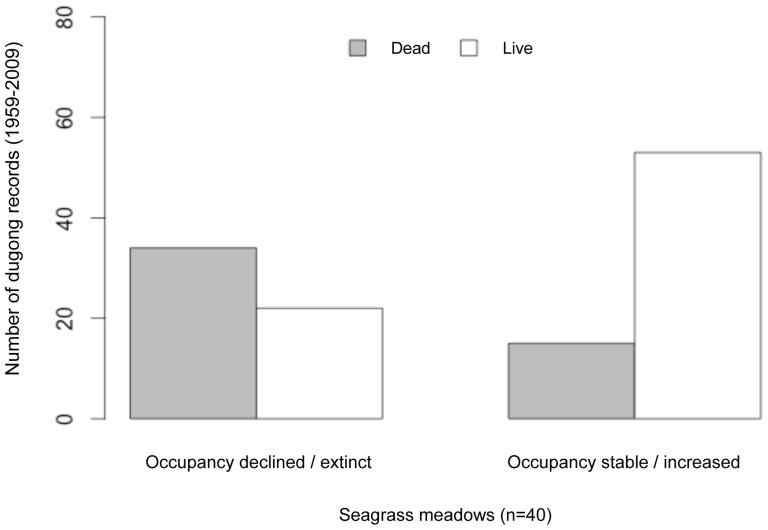
Differences in dugong mortality records at seagrass meadows (n = 40) over time, showing decline in occupancy or persistence. The causes of mortality (including shore-stranded or live-caught individuals in fisheries) recorded were mainly entanglement in gillnets and hunting. Live sightings are recorded both from free-ranging and stranded animals.

Dugong occupancy was higher at meadows around clusters of islands (e.g. Ritchie’s Archipelago, south Andaman, central Nicobar; mean ψ  =  0.30 (SD 0.14)) than at meadows at relatively distant islands (Little Andaman, Car Nicobar; mean ψ  =  0.02 (SD 0.0005), Figure 1, Figure 2). Overall occupancy (mean ψ  =  0.28, range 0.17–0.45) over primary (15-year) periods was positively influenced by meadow sheltering but not by the depth of seagrass beds (Table 2). Detectability (mean *p  = *0.24) ranged between 14–30%. Colonization probabilities ranged between 20–43% across meadows. Persistence probabilities declined considerably through time (c. 55%; Table 1). Dugong occupancy across the archipelago also showed significant reduction (by 60%; from 0.45 to 0.18), especially from 1991 onwards (Table 2, Table 3). Long-term declines in dugong occupancy were correlated with the recorded magnitude of mortality. Sites where dugongs were estimated absent had more past records of gillnet catches and legal hunting by indigenous tribes, as compared to live-sightings (Figure 3). Illegal hunting by settlers might also have probably contributed to declines, but these cases were seldom documented in past reports. Owing to such unavoidable gaps in the data, the proportion of decline in occupancy is at best a conservative estimate.

**Table 2:** Parameter estimates from selected best Bayesian hierarchical models for long-term occupancy dynamics (historical data), with covariates influencing occupancy (zero not included in credible intervals) in bold.

Parameter	Description	Mean	Standard deviation	Credible interval (2.5pc)	Credible interval (97.5pc)
ψ [1]	Overall occupancy for time-period 1	0.175	0.049	0.089	0.28
ψ [2]	Overall occupancy for time-period 2	0.452	0.074	0.311	0.60
ψ [3]	Overall occupancy for time-period 3	0.228	0.062	0.119	0.36
φ [1]	Persistence probability from time-period 1 to time-period 2	0.558	0.156	0.250	0.84
φ [2]	Persistence probability from time-period 2 to time-period 3	0.150	0.078	0.0329	0.33
γ [1]	Colonization probability from time-period 1 to time-period 2	0.429	0.082	0.274	0.59
γ [2]	Colonization probability from time-period 2 to time-period 3	0.292	0.090	0.132	0.48
*p* [1]	Detection probability for time-period 1	0.259	0.089	0.106	0.45
*p* [2]	Detection probability for time-period 2	0.143	0.046	0.066	0.25
*p* [3]	Detection probability for time-period 3	0.231	0.082	0.094	0.41
*alpha*	Intercept of global occupancy model	–4.061	1.73	–7.91	–1.14
*b* [2]	Effect of partially exposed meadow on occupancy relative to exposed meadow	2.11	1.59	–0.79	5.66
*b* [3]	Effect of sheltered meadow on occupancy relative to exposed meadow	4.256	1.92	0.745	8.23
*c* [2]	Effect of depth (high) on occupancy, relative to depth (low)	0.536	1.26	–1.81	3.13
τ	Precision term for group random effects	171.3	387.6	0.163	1248

Note: *b* [1] and *c* [1] were assigned ‘zero’ to mark a clear reference for respective covariates.

**Table 3:** Parameter estimates from selected best Bayesian hierarchical models for current occupancy dynamics, with covariates influencing occupancy (zero not included in credible intervals) in bold.

Parameter	Description	Mean	Standard deviation	Credible Interval (2.5pc)	Credible Interval (97.5pc)
ψ [1]	Overall occupancy for year 1	0.179	0.022	0.135	0.22
ψ [2]	Overall occupancy for year 2	0.089	0.056	0.012	0.22
ψ [3]	Overall occupancy for year 3	0.091	0.057	0.014	0.23
φ [1]	Persistence probability from year 1 to year 2	0.276	0.225	0.009	0.834
φ [2]	Persistence probability from year 2 to year 3	0.599	0.273	0.054	0.983
γ [1]	Colonization probability from year 1 to year 2	0.049	0.046	0.0013	0.171
γ [2]	Colonization probability from year 2 to year 3	0.041	0.041	0.001	0.147
*p* [1,1]	Detection probability for observer 1 for year 1	0.64	0.156	0.318	0.905
*p* [1], [2]	Detection probability for observer 1 for year 2	0.63	0.192	0.228	0.94
*p* [1], [3]	Detection probability for observer 1 for year 3	0.41	0.159	0.125	0.731
*p* [2], [1]	Detection probability for observer 2 for year 1	0.33	0.14	0.091	0.627
*p* [2,2]	Detection probability for observer 2 for year 2	0.53	0.183	0.177	0.863
*p* [2], [3]	Detection probability for observer 2 for year 3	0.77	0.139	0.448	0.968
*E* [1,1]	False positive detection probability for observer 1 for year 1	0.03	0.0177	0.0055	0.0711
*E* [1], [2]	False positive detection probability for observer 1 for year 2	0.075	0.0173	0.036	0.0989
*E* [1], [3]	False positive detection probability for observer 1 for year 3	0.04	0.023	0.0074	0.0916
*E* [2], [1]	False-positive detection probability for observer 2 for year 1	0.023	0.0165	0.0055	0.0677
*E* [2,2]	False-positive detection probability for observer 2 for year 2	0.024	0.017	0.0055	0.0687
*E* [2], [3]	False-positive detection probability for observer 2 for year 3	0.021	0.015	0.0054	0.0611
*q1* [2]	Effect of partially exposed meadow relative to sheltered meadow	5.12	5.59	–2.656	19.22
*q1* [3]	Effect of exposed meadow relative to sheltered meadow	–28.98	18.63	–73.61	–2.99
*q2* [2]	Effect of Sc2 relative to Sc1	1.55	3.60	–4.73	9.72
*q2* [3]	Effect of Sc3 relative to Sc1	7.79	13.74	–15.19	39.08
*q2* [4]	Effect of Sc4 relative to Sc1	–21.97	12	–49.67	–4.52
*q3* [2]	Effect of meadows arrived recently	–30	20.34	–76.09	1.98
*q3* [3]	Effect of persistent meadows	41.23	17.11	12.9	78.22
*q3* [4]	Effect of dynamic meadows	13.25	7.41	2.76	30.75
τ	Precision term for group random effects	0.001	0.003	0.00003	0.0070

Note: *q1* [1], *q2* [1], *q3* [1] were assigned ‘zero’ to mark a clear reference point for respective covariates. Sc refers to categorical variable ‘seagrass species composition’ (see methods).

### Short-term dynamics

Individual dugong encounter-rates, based on direct sightings were low (seven individuals seen in six meadows over three years), and locations where dugongs persist might only have a few individuals. Sightings of calves are also rather uncommon (two calves in the last three years). Detections mostly corresponded to dugong feeding trails observed in seagrass meadows and a few additional direct sightings by key informants. Long-term mean occupancy probability was not related to current occupancy at a location, suggesting that occupancy at a meadow might be influenced more by recent factors. Dugong occupancy (ψ) across the entire archipelago declined from 0.18 in 2010 (first year of sampling) to 0.09 in the next two years. Detection probabilities were similar between feeding trail surveys (mean *p* = 0.57, 0.41–0.65) and direct sightings/informant reports (mean *p* = 0.53, 0.33–0.77) (Table 2). These were nearly 2.5 times higher than estimated *p* for historical occurrence records. Persistence probabilities were 60% (SD = 27%) between years, and annual colonization rates were low (3–5%) indicating persistence of dugongs at the same meadows in the short-term (Table 3). Current dugong occupancy was positively influenced by the presence of sheltered meadows dominated by *Halophila* sp. and *Halodule pinifolia*, suggesting persistence at least between 1995 and 2010 (Table 3). Occupancy appears to have been stable in three regions: Ritchie’s Archipelago, Central Nicobars and South Andaman (0.13–0.56). Major declines were estimated from north Andaman (from about 25% to 0.10%), Little Andaman (5% to 0.01%) and Little and Great Nicobars (20% to 0.06%) (Table 2, Table 3; Figure 1, Figure 2). It is unclear if dugongs occurred even in the past around Car Nicobar.

### Ecological and anthropogenic variables affecting current dugong occupancy

**Figure 4:**
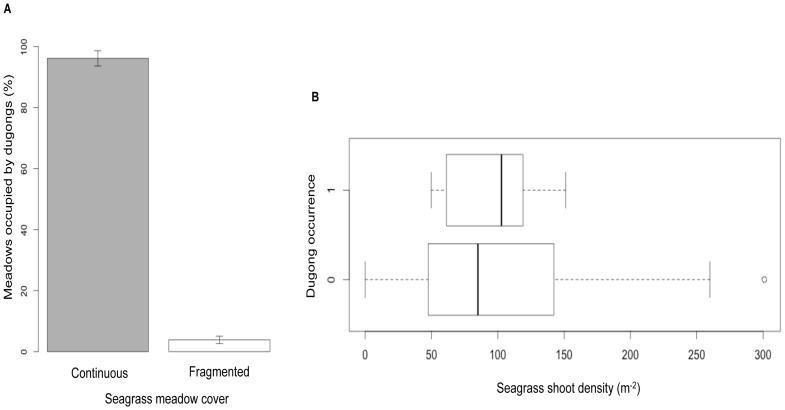
Dugong occurrence in relation to seagrass meadow cover and shoot density. a) Dugongs were not found in patchy, fragmented meadows with low seagrass cover (data available for n = 20 meadows out of 57). Error bars indicate standard deviation about estimated mean occupancy. b) Variations about median shoot densities of *Halophila* and *Halodule* spp. in seagrass meadows maintained by dugong grazing, and those without dugong grazing (data available for n = 14 of 57 meadows).

**Figure 5:**
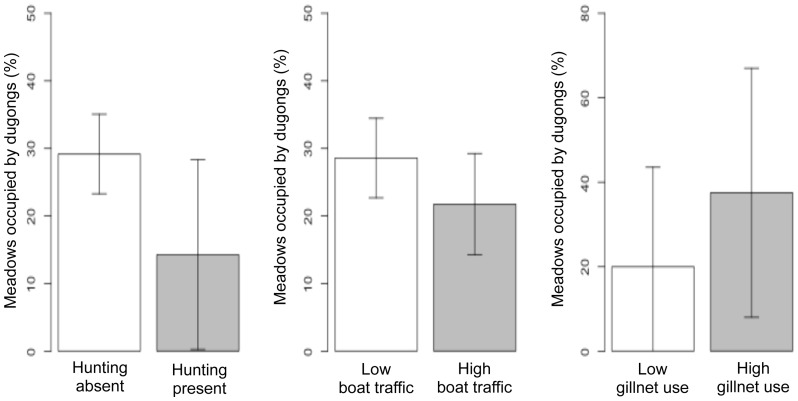
Overlaps in distribution of dugongs and anthropogenic threats. a) Dugong occupancy (ψ) was lower in locations with prevalence of hunting, b) meadows with dugongs and without dugongs had almost similar boat traffic (5+ boats d-1) and c) dugong distribution overlapped with high gillnet usage. Error bars indicate standard deviation about the mean occupancy (for n = 38 out of total 57 meadows).

Dugongs appeared to avoid patchy meadows with low seagrass cover (Figure 4A). Meadows where dugongs were present had lower variance in shoot densities of *Halophila* sp. and *Halodule pinifolia* than in meadows where dugongs were absent (Figure 4B). Dugong occurrence was higher in areas where hunting was low or absent (Figure 5). However, meadows with dugong occurrence also had considerable gillnet use and boat-traffic levels (Figure 5). Therefore, it is likely that these threats continue to affect dugongs in meadows where they persist, but the current study is only able to suggest them as factors of potential significance for dugong declines.

## Discussion

Our results indicate that dugongs in the Andaman and Nicobar archipelago have declined significantly (about 60%) in their occupancy over the last two decades. It is uncertain if dugong populations can sustain such declines in spatial distribution. This trend of decline is similar to cases of dugong declines in many seascapes in south and southeastern Asia [20], [23], [56]. The occupancy framework we employed provides a conservative yet useful approach to assess long-term distributional dynamics from ‘imperfect’ and fragmentary long-term occurrence records that include false negatives. This approach can also be effectively used to identify factors influencing current patterns of persistence and local extinction.

We use the Bayesian approach to make best use of the typically fragmentary data available for rare, vulnerable species like the dugong, with minimal data available. Given the patchiness of data and evident differences in the robustness of different data sources, we employed a relatively conservative approach to modeling population trends, treating such information as ‘weak or suggestive’ in the Bayesian framework. We have taken care to make the ecological criteria for model assumptions explicit to specify bounds to the interpretation of these results. Though we appreciate that there is no substitute for ‘robust’ data with large sample sizes, in reality data on rare species may be very difficult to obtain, even with the most detailed surveys and robustly designed methods. In some such cases the methods presented may be useful to conservationists and ecologists to quantitatively estimate long-term trends in species’ distribution using past occurrence records.

Dugong populations are currently restricted to a few areas where they seem to have persisted over several years. These locations typically have sheltered and continuous seagrass meadows dominated by short-lived species. Dugongs are known to incidentally consume invertebrates along with seagrasses, and sometimes selectively feed on ascidians and polychaetes, possibly due to nutritional stress caused by seasonality in seagrasses [57]. However, seagrasses still form the dominant diet (about 74% documented by Preen, 1995) of dugongs and it is unlikely that other food items alone will enable dugongs to persist despite loss of seagrasses [58]. Seagrass shoot densities of occupied meadows in the A&N archipelago were more homogeneous than unoccupied meadows with similar species composition, indicating that continued dugong grazing might be maintaining these meadows with pioneer, fast-growing seagrass species [30], [59], [60]. Dugong declines will lead to reduction in herbivory, which along with factors such as altered coastal sedimentation levels, could accelerate seagrass succession towards fibrous species or make meadow cover patchy. This may in turn make meadows unavailable to recolonizing dugongs. We did not find dugongs in patchy meadows with low seagrass cover. Meadow fragmentation could be linked to sedimentation-related burial caused by seasonal storms or anthropogenic factors (e.g. increased coastal development/land-use practices) [61]. The volcanic origin of the A&N archipelago [33] makes sedimentation a key process in coastal waters. Understanding its effects on seagrass cover and composition could help understand patterns of dugong meadow use [62], [63].

Importantly, dugong occupancy across the A&N archipelago did not appear to be limited by the availability of seagrass habitats. Yet, from island groups such as the Little and Great Nicobars, we were unable to detect dugongs even once in current surveys, despite regular past sightings and the continued persistence of extensive meadows. At these locations dugongs are most likely locally extinct. These observations suggest that dugong declines in the A&N archipelago may have been possibly driven by anthropogenic factors. These factors include incidental mortality from entanglement in gillnets and targeted hunting (both legal and illegal, i.e. by indigenous people and settlers). Other factors such as mortality from boat propeller strikes or the 2004 tsunami, although not assessed here, could be important in causing recent declines in occupancy. Our recent observations further highlight that these threats continue to negatively affect dugongs in the A&N archipelago.

Incidental by-catch could have been an important reason behind dugong mortality and [61], [64] conservation measures will need to involve regulation of gillnetting practices in meadows used by dugongs. Additionally, the issue of legal hunting by indigenous tribes needs to be addressed with culturally sensitive and inclusive approaches. Dugongs have the highest protection status in Indian wildlife conservation law [65] but the A&N tribes are legally exempt due to the significant totemic and heritage value they place on the dugong. In the southern Nicobars, local extirpations (despite presence of extensive meadows) are probably linked to the long present ritual hunting of dugongs by these tribes [26]. Such hunting by indigenous tribes [66], [67], (though legal) can negatively affect dugong populations, if it does not follow practices of sustainable harvest [68]. It is important to motivate these indigenous groups to implement self-imposed voluntary bans on hunting; or to set harvest thresholds over time periods that may allow local population recovery to take place [66], [70]. Illegal hunting by non-indigenous groups may be equally serious, if not more so, but is significantly under-reported and difficult to monitor. Such hunting needs to be banned through strong enforcement, to protect remaining populations of dugongs occupying settler-dominated areas. Reduction of hunting pressure has shown encouraging recoveries in dugong populations in the Arabian Gulf [69], and highlights the importance of tackling the issue within this region.

Although few studies exist on long-term occupancy dynamics of marine mammals, evaluations of occupancy trends for other rare, wide-ranging terrestrial species indicate that drastic reductions can seriously increase the risk of regional extinction (e.g. threatened large mammals [70], [71]; the endangered Spotted Owl [10]; amphibians [9], [36] and long-lived plants [72]). Conserving rare and elusive marine mammal populations in logistically challenging locations is fraught with difficulties. While our models provide support for considerable declines, it is difficult to determine the specific contribution of ecological and anthropological drivers of this decline without strong direct information on hunting patterns, human disturbance, or dugong local abundance, movements and seagrass dynamics. However, our study makes a first attempt at identifying which of these factors are likely important in driving local persistence and extinction of dugongs. These findings, while admittedly open to further detailed investigation, can help in spatially prioritizing dugong conservation efforts in the A&N archipelago.

Despite the evident difficulties in conserving this population, concerted efforts with multi-pronged conservation approaches may offer opportunities to improve protection of the dugong within the northern Indian Ocean [73]. In data-poor situations, we believe that our study could be relevant to similar situations across the world, both for dugongs and other marine mammals across large spatial scales. The study highlights the value of combining historical and current data from all available sources to identify factors underlying long-term distributional trends in elusive, threatened marine mammal populations.

## Supporting Information

Appendix S1 **Locations sampled for dugong occupancy surveys in the Andaman and Nicobar Islands.** Key: **Andaman Islands:** 0-East I., 1-Landfall I., 2-Reef I., 3-Radhanagar, 4-Paget I., 5-Casuarina Bay, 6-Temple Island, 7-Ross Smith beachfront, 8-Craggy-Kalipur, 9-Atlanta Bay,10-North Reef I., 11-La’touche, 12-Stewart I., 13-Sound I., 14-Mayabunder Bay, 15-Austen Harbour, 16-Interview I., 17-Long I., 18-North Button, 19-Strait I.,20-Henry Lawrence I., 21-John Lawrence I., 22-Havelock Fusilier Channel, 23-Radhanagar, 24-Point, 25-Neil, 26-Hugh Ross, 27-Shoal Bay, 28-Port Blair 1, 29-Port Blair 2, 30-North Bay & Mt. Harriett, 31-Rutland, 32-Burmanullah, 33-Chidiyatapu, 34-Cinque I., 35-Kanaidera, 36-Mahuadera, 37-Tarmugli, 38-MGMNP, 39-Dugong Creek, 41-Butler Bay, 42-Hut Bay, 43-West Bay, 44-Ekiti; **Nicobar Islands:** 0-South+West, 1-North+East, 2-Teressa, 3-Flotsam, 4-Police camp, 5-Trinket, 6-Trinket, 7-Champian, 8-Hitui, 9-Malacca, 10-Kardip, 11-Altaiyak, 12-Camorta NW,13-Derring Bay, 14-West, 15-Bada Enaka, 16-Marine, 17-West Bay, 18-North Bay, 19-North, 20-Pilomillow, 21-Campbell Bay, 22-Casuarina Bay, 23-Bquarry, 24-Laxminagar, 25-Laful Bay.
(DOCX)

Appendix S2 (DOCX)
