# Supplementary material for: Long-Term Occupancy Trends in a Data-Poor Dugong Population in the Andaman and Nicobar Archipelago
Source: PLoS One. 2013 Oct 15;8(10):e76181. doi: 10.1371/journal.pone.0076181 (PMC3797053; doi:10.1371/journal.pone.0076181)
Supplement: Appendix S1 — Locations sampled for dugong occupancy surveys in the Andaman and Nicobar Islands. Key: Andaman Islands: 0-East I., 1-Landfall I., 2-Reef I., 3-Radhanagar, 4-Paget I., 5-Casuarina Bay, 6-Temple Island, 7-Ross Smith beachfront, 8-Craggy-Kalipur, 9-Atlanta Bay,10-North Reef I., 11-La’touche, 12-Stewart I., 13-Sound I., 14-Mayabunder Bay, 15-Austen Harbour, 16-Interview I., 17-Long I., 18-North Button, 19-Strait I.,20-Henry Lawrence I., 21-John Lawrence I., 22-Havelock Fusilier Channel, 23-Radhanagar, 24-Point, 25-Neil, 26-Hugh Ross, 27-Shoal Bay, 28-Port Blair 1, 29-Port Blair 2, 30-North Bay & Mt. Harriett, 31-Rutland, 32-Burmanullah, 33-Chidiyatapu, 34-Cinque I., 35-Kanaidera, 36-Mahuadera, 37-Tarmugli, 38-MGMNP, 39-Dugong Creek, 41-Butler Bay, 42-Hut Bay, 43-West Bay, 44-Ekiti; Nicobar Islands: 0-South+West, 1-North+East, 2-Teressa, 3-Flotsam, 4-Police camp, 5-Trinket, 6-Trinket, 7-Champian, 8-Hitui, 9-Malacca, 10-Kardip, 11-Altaiyak, 12-Camorta NW,13-Derring Bay, 14-West, 15-Bada Enaka, 16-Marine, 17-West Bay, 18-North Bay, 19-North, 20-Pilomillow, 21-Campbell Bay, 22-Casuarina Bay, 23-Bquarry, 24-Laxminagar, 25-Laful Bay. (DOCX) [file pone.0076181.s001.docx]

**Supporting Information S1**

**Figure S1.**


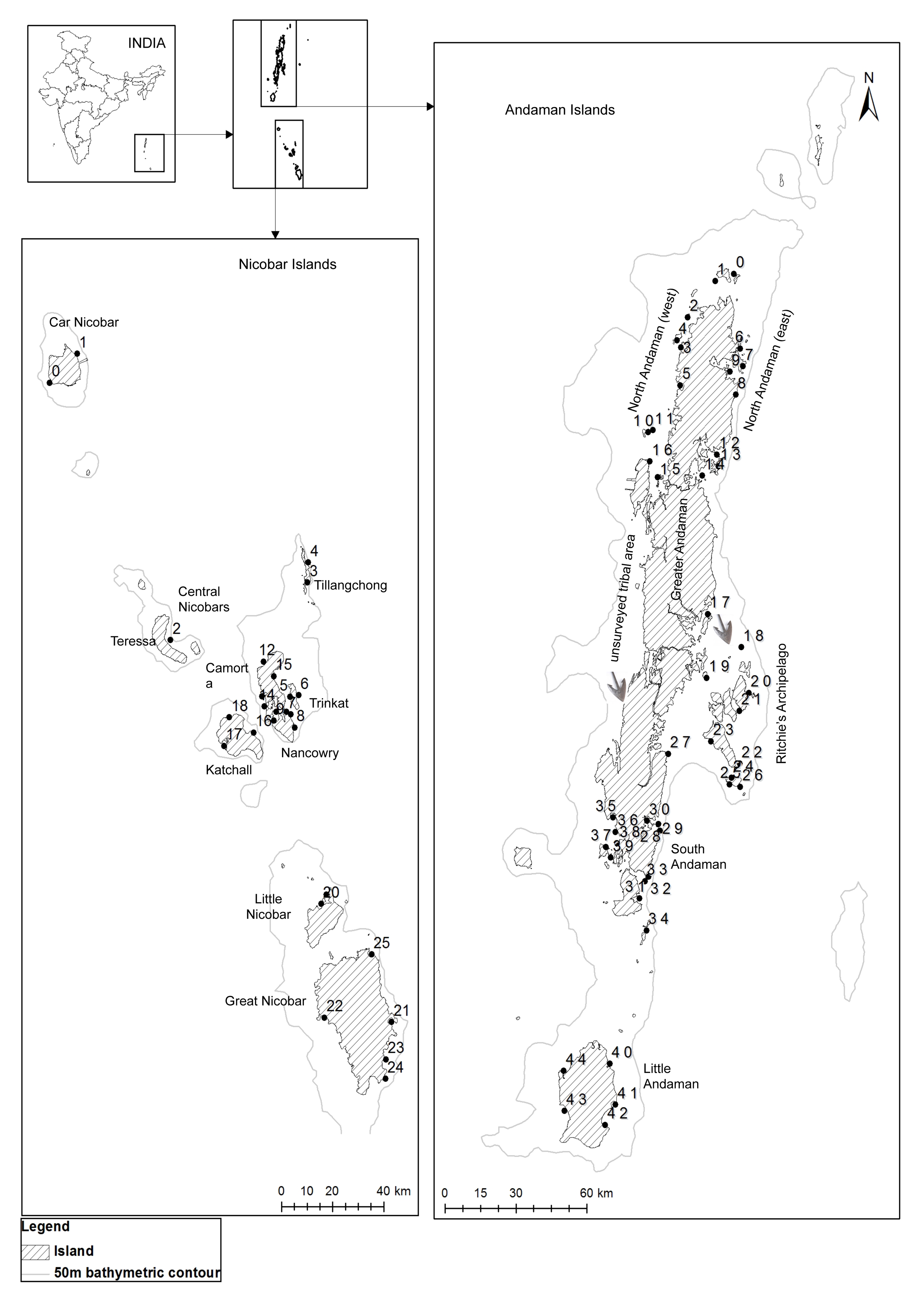


**Key:** **Andaman Islands:** 0-East I., 1-Landfall I., 2-Reef I., 3-Radhanagar, 4-Paget I., 5-Casuarina Bay, 6-Temple Island, 7-Ross Smith beachfront, 8-Craggy-Kalipur, 9-Atlanta Bay,10-North Reef I., 11-La’touche, 12-Stewart I., 13-Sound I., 14-Mayabunder Bay, 15-Austen Harbour, 16-Interview I., 17-Long I., 18-North Button, 19-Strait I.,20-Henry Lawrence I., 21-John Lawrence I., 22-Havelock Fusilier Channel, 23-Radhanagar, 24-Point, 25-Neil, 26-Hugh Ross, 27-Shoal Bay, 28-Port Blair 1, 29-Port Blair 2, 30-North Bay & Mt. Harriett, 31-Rutland, 32-Burmanullah, 33-Chidiyatapu, 34-Cinque I., 35-Kanaidera, 36-Mahuadera, 37-Tarmugli, 38-MGMNP, 39-Dugong Creek, 41-Butler Bay, 42-Hut Bay, 43-West Bay, 44-Ekiti; **Nicobar Islands:** 0-South+West, 1-North+East, 2-Teressa, 3-Flotsam, 4-Police camp, 5-Trinket, 6-Trinket, 7-Champian, 8-Hitui, 9-Malacca, 10-Kardip, 11-Altaiyak, 12-Camorta NW,13-Derring Bay, 14-West, 15-Bada Enaka, 16-Marine, 17-West Bay, 18-North Bay, 19-North, 20-Pilomillow, 21-Campbell Bay, 22-Casuarina Bay, 23-Bquarry, 24-Laxminagar, 25-Laful Bay
