# Supplementary material for: Long-Term Occupancy Trends in a Data-Poor Dugong Population in the Andaman and Nicobar Archipelago
Source: PLoS One. 2013 Oct 15;8(10):e76181. doi: 10.1371/journal.pone.0076181 (PMC3797053; doi:10.1371/journal.pone.0076181)
Supplement: Appendix S2 — (DOCX) [file pone.0076181.s002.docx]

**PONE-D-13-17996**

**Supporting Information S2**

**Appendix S2.**

*Bayesian model specification*

For the dynamic occupancy analysis we used the basic model formulation described in Royle & Dorazio (2008), with modifications suited to our study based on Nichols et al. (2008), Saracco et al. (2011) and Aing et al. (2011). We can assume occupancy probability for each meadow i to follow a uniform prior distribution whose parameter space lies between 0 and 1, as ψ_i_ ~ *Uniform(0,1)*. We used a similar prior for the other parameter of interest (detection probability), as *p*_iwt_ ~ *Uniform(0,1),* where i refer to meadows, w to replicate observations and t are the sampling occasions (time). To model turnover in occupancy with time, we need to estimate survival probability phi and colonization probability gamma, for which *ϕ[t:T-1]~ Uniform(0,1) γ[t:T-1]~Uniform(0,1)* were specified; note that these cannot be estimated for occasion t=1 but only for occasions t=2 and t=3, relative to the occupancy state at the previous occasion. The occupancy process model was specified as a binomial regression model with logit link on occupancy probability *logit*(ψ_i_*)*<-*b0*+*b1***habitat* *covariate[i]*+*spatial effects* and *Z_it_ ~ Bernoulli*(ψ_i_) (where *Z[i,t]* represented the occupancy matrix over time t for all meadows i (see Table for details). The random effects parameter *spatial effects [site.id[i]]* was specified to estimate effects of larger island areas to which adjacent meadows were grouped, on variation in meadow occupancy. The random effects were also specified as an uninformative prior distribution as *spatial effects [k] ~ Normal (0, τ)*; *τ ~ Gamma (0.001, 0.001)*. If the spatial variance were observed to be high (positive), it would indicate large spatial effects, whereas lower values would indicate the presence of only weak spatial effects. If we expected a positive effect of a certain covariate on occupancy, the prior distribution for its effect size (slope *b1*) was specified as *b1 ~ lognormal (a, c)* but high variance. Likewise, priors to indicate expectations of negative slopes were specified with a negative mean (*b1 ~ Normal (-b, d))* and high variance; and for unknown/zero effects, an uninformative prior *b1 ~ Normal (0, 1000)*. The degree of belief in the priors was specified by appropriate precision terms c, d as low (poor information) or high (good information).

The imperfect observation model on the true process of occupancy was given by *muY1*[i,j,1] <- *z*[i,1]**Pr*[i,j,1]; and where *Y*[i,j,t] ~ *Bernoulli (muY1[i,w,t])* represented detection and non-detection data at each replicate j for meadow i at time t. The process model was used for occasion 1 and later subsequently updated for occasions 2 and 3 by the survival and colonization probabilities as: *meanpsi*[t] <- *meanpsi*[t-1]**phi*[t-1]+ (1-*meanpsi*[t-1])**gamma*[t-1]. For these occasions the observation model was given by

*muz*[i,t] <- *z*[i,t-1]**phi*[t-1]+ (1-*z*[i,t-1])**gamma*[t-1]; *z*[i,t] ~ dbern(*muz*[i, t]).

The specification was slightly modified for analysis of current occupancy data (short-term dynamics) by incorporating a probability of false positive detection conditional on observer replicate *w*, *E*[w] for potential false recording of dugongs by local informants (observer replicate w=2). The observation model was changed to *muY[i,w,t] <- Z_it_. P_iwt_ + (1-Z_it_). E_iwt_* for meadow i, detected by observer w at time t. The prior probability of *E*[w] was constrained to take low values (as expected of misidentification of dugongs, up to 5%) such that *p*>>*E* (*E*[w] ~ dbeta(1,20)) for parameter identifiability (Aing et al., 2011). For the current model, two different observation methods (feeding signs, direct sighting by local informer) were used as survey replicates at each meadow. For parameter estimation, we used 15000 to 20000 Markov Chain Monte Carlo (MCMC) simulations with a discarded burn-in of 1000 iterations, for 2 Markov chains, with 1 to 2 thins each.

Table S1. Parameter and data definitions for historical and current occupancy.

| **Model parameter** | **Definition** |
| --- | --- |
| (psi) | Occupancy probability: probability of dugong presence, i.e. (proportion of sites occupied) |
|  | Detection probability: Detection of feeding sign or direct sighting given that dugongs were present and available for detection |
| (phi) | Survival probability: Probability that a meadow remains occupied from time-period 1 to time-period 2 |
| (gamma) | Colonization probability: Probability that a meadow becomes occupied in time-period 2 from an unoccupied state in time-period 1 |
|  (E) | False-positive detection probability: probability that a sign or sighting is reported falsely as an index of dugong presence |
| a, b, c, d | Intercept and slope parameters on meadow-level ecological covariates affecting historical (long-term) occupancy dynamics |
| q1, q2, q3 | Intercept and slope parameters for meadow-level ecological covariates affecting short-term occupancy dynamics |
| (tau) | Precision (1/Variance) of random effects term showing island group effects on individual meadow occupancy |
|  | |
| **Data** | **Definition** |
|  | Matrix of dugong detection (1) and non-detection (0) in meadow i for observer j (replicate) at time t |
|  | Matrix of dugong detection (1) and non-detection (0) in meadow i at time t |
|  | Variable indexing observer; w=[0,1] where 0 indexes feeding sign-based detection and 1 indexes local observer report of direct sighting. |
|  | Habitat covariate measured at level of site i |
|  | Index referring to membership of meadow i to a larger island group |
